# Nomogram for preoperative estimation risk of cervical lymph node metastasis in medullary thyroid carcinoma

**DOI:** 10.3389/fonc.2022.883429

**Published:** 2022-10-12

**Authors:** Zhiyan Luo, Yurong Hong, Caoxin Yan, Qin Ye, Yong Wang, Pintong Huang

**Affiliations:** ^1^ Department of Ultrasound Medicine, Second Affiliated Hospital, School of Medicine, Zhejiang University, Hangzhou, China; ^2^ Department of Pathology, Second Affiliated Hospital, School of Medicine, Zhejiang University, Hangzhou, China; ^3^ Department of Surgery, Second Affiliated Hospital, School of Medicine, Zhejiang University, Hangzhou, China

**Keywords:** sporadic medullary thyroid carcinoma, cervical lymph node metastasis, ultrasonography, prevention lymph node dissection, nomogram

## Abstract

**Objectives:**

Cervical lymph node metastasis (CLNM) is common in medullary thyroid carcinoma (MTC), but how to manage cervical lymph node involvement of clinically negative MTC is still controversial. This study evaluated the preoperative features and developed an ultrasound (US)-based nomogram to preoperatively predict the CLNM of MTC.

**Materials and methods:**

A total of 74 patients with histologically confirmed MTC were included in this retrospective study and assigned to the CLNM-positive group and CLNM-negative group based on the pathology. The associations between CLNM and preoperative clinical and sonographic characteristics (size, location, solid component, shape, margin, echogenicity, calcification, and extracapsular invasion of the tumor) were evaluated by the use of univariable and multivariable logistic regression analysis. A nomogram to predict the risk of the CLNM of MTC was built and assessed in terms of discrimination, calibration, and clinical usefulness.

**Results:**

The nomogram was based on three factors (tumor margin, US-reported suspicious lymph node, and extracapsular invasion US features) and exhibited good discrimination with an area under the curve (AUC) of 0.919 (95% CI, 0.856–0.932). The calibration curves of the nomogram displayed a good agreement between the probability as predicted by the nomogram and the actual CLNM incidence.

**Conclusions:**

We constructed and validated a US-based nomogram to predict the risk of CLNM in MTC patients, which can be easily evaluated before surgery. This model is helpful for clinical decision-making.

## Introduction

Medullary thyroid carcinoma (MTC) is a rare disease, amounting to about 2%–5% of all thyroid malignancies globally (1–3). It is characterized by a relatively slow tumor growth but early lymph node (LN) metastasis (LNM), which appeared in 40.0%~66.7% of patients when initially diagnosed, with the predominance of cervical lymph node metastasis (CLNM) (4). In sporadic MTC, the proportion of central and lateral neck LNMs related with T1 tumors is 14% and 11%, respectively; the proportion is 86% and 93%, respectively, with pT4 tumors (5). Palpable thyroid nodules are associated with a 70% rate of CLNM and a 10% rate of distant metastasis (6).

Total thyroidectomy (TT) and dissection of cervical LNs are standard approaches for MTC in light of preoperative serum calcitonin (Ctn) levels, ultrasound (US)-reported suspicious LN findings, and intraoperative or fine-needle aspiration (FNA)-proven CLNM (1, 7, 8). However, there is a disputed topic in performing lateral neck dissection (LND) in patients without evidence of CLNM on preoperative US. More aggressive prophylactic LND may raise the risk of severe nerve injury and hypoparathyroidism without obvious survival benefits (9).

Preoperative imaging plays an important role in the diagnosis and staging of MTC. Although several studies have reported high-risk factors relative to clinical and US features predictive of CLNM in MTC (10–12), the results have been conflicting. In addition, some of the risk factors identified, such as TNM stage, are only available after the operation (13, 14) and cannot help in determining the extent of thyroid surgery. Seeking a suitable and noninvasive approach for evaluating CLNM is therefore of great importance.

For this reason, we constructed and validated a nomogram to predict CLNM based on clinical and US features, a precise, simple, and objective scoring system for preoperatively quantifying the probability of CLNM.

## Materials and methods

**Figure 1:**
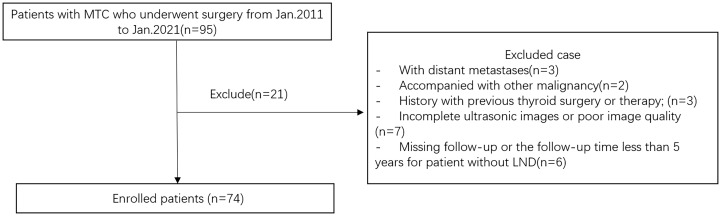
Flowchart of the patients enrolled in our study. MTC, medullary thyroid carcinoma; LND, lymph node dissection.

This retrospective study was approved by the Ethics Committee of the Second Affiliated Hospital of Zhejiang University School of Medicine, and the requirement for informed consent was waived. We retrospectively evaluated the preoperative clinical and US features for predicting CLNM in patients with pathologically confirmed MTC surgery in our hospital between January 2011 and January 2021. The inclusion criteria of the nodules were as follows: 1) the thyroid US examination was carried in our department within 2 weeks before surgery; 2) patients who underwent initial thyroid surgery with central neck dissection (CND) or modified radical neck dissection during the initial surgery and were pathologically confirmed as MTC; 3) no other treatment before surgery. The exclusion criteria were as follows: 1) patients with distant metastases or accompanied by other malignancies; 2) incomplete or unqualified ultrasound images; 3) patients are treated by chemotherapy or radiotherapy before surgery; 4) for MTC patients without LND, regularly followed up for less than 5 years. According to the above criteria, 74 patients (31 men and 43 women, mean age 43.2 ± 10.9 years) were enrolled. **Figure 1** showed the flowchart of the patients enrolled in our study. All patients underwent TT with bilateral CND, 54 (72.9%) patients underwent modified radical neck dissection during the initial surgery, and the remaining 20 MTC patients without LND were regularly followed up for at least 5 years, with a median of 6.7 years [interquartile range (IQR) 5.0–9.3 years]. Among the study subjects, 33 (44.6%) patients were placed in the LNM-negative group and 41 (55.4%) patients were placed in the CLNM-positive group according to the pathology results. All cases were regularly followed.

### Requirements for ultrasound images

The US images obtained from the picture archiving and communication system (PACS) workstations should contain the following requirements: 1) including as many malignant characteristics of the tumor as possible in the longitudinal and transverse planes; 2) clearly exhibiting the extent of contact with the adjacent capsule; 3) US findings, including the size, location, solid component, shape, aspect ratio (A/T), margin, echogenicity, echotexture, peripheral halo sign, extracapsular invasion, calcifications, vascularization, suspicious metastatic LNs, Contrast-Enhanced Ultrasound (CEUS) patterns, and elastic scores were independently evaluated, as previously reported (15) by two sonographers with more than 10 years of experience in thyroid US and were blinded to the clinical outcome. In patients with multifocal MTCs, the dimensions of the largest MTC lesion were used. Tumor size was classified according to the maximum diameter. Tumor shape was classified as either oval and round or irregular. The A/T was classified as <1 or ≥1. The internal echogenicity was categorized as hyperechogenicity, isoechogenicity, hypoechogenicity, or marked hypoechogenicity compared with the adjacent cervical muscle. Margins were classified as smooth, lobulated, microlobulated, and spiculated. Calcifications, if present, were classified as microcalcifications, macrocalcifications, or mixed calcifications. Tumor vascularity was assessed by color Doppler flow imaging (CDFI) and classified according to the Adler criteria [16] from 0 to 3. The presence of extracapsular invasion (defined as that the tumor in contact with the adjacent capsule, so that the continuity of the capsule line was interrupted or covered by lesions). In the preoperative assessment of CLNMs, a suspicious LN exhibited the following features: internal microcalcification, loss of hilar echogenicity, exhibition of peripheral flow, and cystic or hyperechoic change. Elastography images were classified according to the scores by Hong et al. (15) into a score of 1–6. In this study, a malignant lesion showing Hong scores of 4–6 was considered as “hard” malignancy and the remaining scores as “soft.” The CEUS patterns of the thyroid nodules were classified as hyperenhancement and hypoenhancement.

The clinical characteristics, including gender, age, preoperative Ctn, and carcinoembryonic antigen (CEA), were collected from the electronic medical records. In line with a previous research (16), the thresholds set for Ctn and CEA were as follows: Ctn ≥8.4 pg/ml (men), Ctn ≥5.0 pg/ml (women), and CEA ≥5 ng/ml.

### Statistical analysis

The statistical analyses were performed through SPSS version 16.0 (SPSS Inc., Chicago, IL, USA). Continuous data are presented as mean ± standard deviation (SD) and compared by using Student’s t test. Categorical data were compared using the Pearson chi-square test and Fisher’s exact test, and the receiver operating characteristic (ROC) curve analysis was performed to determine the optimal cutoff points for tumor size in US. Variables that proved to be statistically significant on univariate analysis were included in the multivariate logistic regression to evaluate risk factors for CLNM in MTC patients.

A nomogram was built according to the results of the binary logistic regression to assess the risk of CLNM preoperatively by using R software (version 3.5.1). The performance of the nomogram was further evaluated by discrimination and calibration. ROC was employed to test the discriminative power and consensus of our formulated CLNM prediction model. The calibration of the prediction model was carried out by plotting the CLNM-positive predicted probability of the nomogram against the observed probability. In addition, the nomogram was subjected to 1000 bootstrap resamples for internal validation to assess the accuracy of the constructed logistic regression model.

## Results

### Demographic and clinicopathologic characteristics

All 74 MTC patients, including 31 men and 43 women, were confirmed by surgery and pathology. Among the 74 eligible patients, 41 patients had CLNM [10 patients with central compartment lymph node metastasis (CCLNM), four patients with lateral compartment lymph node metastasis (LCLNM), and 27 patients with both compartment LNM]; the remaining 33 patients showed negative CLNM. Sixty-four patients had measurements of their serum Ctn levels and 62 had measurements of their CEA. In the ROC analysis, the optimal cutoff tumor size in US between the two groups was 2.19 cm [area under the curve (AUC), 0.579; 95% confidence interval (CI), 0.449–0.709]. The baseline characteristics of the patients in each group are presented in **Table 1**. The level of preoperative serum Ctn was significantly higher in the CLNM-positive group than that in the CLNM-negative group (*p* = 0.001); no significant differences were found between the two groups in terms of age, gender, tumor location, or CEA.

**Table 1:** Clinical and US imaging characteristics of MTC.

Characteristics	Non-LNM group	LNM group	χ^2^	*p*
Mean age	46.85 ± 14.17	48.95 ± 13.43	0.075	0.785
Preoperative Ctn	270.66 ± 426.32	549.1 ± 697.94	12.47	0.001
Preoperative CEA	103.11 ± 224.35	113.2 ± 232.85	0.052	0.863
Sex			0.007	0.934
Men	14	17		
Women	19	24		
Location 1			2.387	0.122
Left lobe	15	26		
Right lobe	18	15		
Location 2			0.129	0.937
Upper lobe	12	16		
Middle lobe	17	21		
Lower lobe	4	4		
Nodule size			5.734	0.017
≤2.19	4	26		
>2.19	29	15		
Aspect ratio			1.471	0.225
≤1	28	33		
>1	5	11		
Shape			4.062	0.044
Oval and round	19	14		
Irregular	14	27		
Margin			23.467	0
Smooth	6	1		
Lobulated	17	6		
Microlobulated	4	5		
Spiculated	6	29		
Extracapsular invasion			28.48	0
Yes	6	33		
No	27	8		
Echogenicity			1.958	0.376
Markedly hypoechogenic	18	27		
Hypoechogenic	14	14		
Hyperechoic or Isoechoic	1	0		
Composition			0.498	0.48
Solid	28	37		
Mixed	5	4		
Echotexture			2.02	0.155
Homogeneous	6	3		
Heterogeneous	27	38		
Calcification			0.485	0.785
Absent	13	13		
Microcalcification	14	20		
Macrocalcification	6	8		
Peripheral halo sign			1.517	0.218
Absent or fine halo	30	39		
Thick or irregular halo	3	2		
Vascularity			0.659	0.883
0	6	6		
1	6	8		
2	3	6		
3	18	21		
Elastic score			1	0.583
<3	1	0		
≥3	13	10		
CEUS patterns			0.012	0.912
Hypoenhancement	8	6		
Hyperenhancement	3	2		
US-reported LN status			27.43	0
Positive	2	27		
Negative	31	14		

US, ultrasound; MTC, medullary thyroid carcinoma; LNM, lymph node metastasis; Non-LNM, non-lymph node metastasis; Ctn, Calcitonin; CEA, carcino-embryonic antigen; CEUS, Contrast-enhanced Ultrasound; LN, lymph node.

### Selected factors for the model

The preoperative sonographic features of MTCs are also described in **Table 1**. After univariable analysis, MTCs with positive CLNM were more often nodules with a larger size (*p* = 0.017), irregular shape (*p* = 0.044), microlobulated or spiculated margin (*p* < 0.001), extracapsular invasion (*p* < 0.001), and US-reported suspicious LN (*p* < 0.001) than those without CLNM. While the variables of echogenicity, solid component, echotexture, presence of calcification, halo sign, vascularity, elastic scores, and CEUS patterns were not significantly different between the two groups (*p* > 0.05 for all).

After multivariate analysis, extracapsular invasion, margin, and US-reported LN status remained independent predictors for CLNM, as shown in **Table 2**.

**Table 2:** Multivariate analysis of risk variables for CLNM of MTC.

Characteristics	β	Odds ratio (95% CI)	*p*
Extracapsular invasion	1.82	1.39-27.72	0.017
US-reported LN status	2.74	2.21-109.61	0.006
Margin	1.32	1.51-9.35	0.004

LN, lymph node.

### Predictive nomogram for the probability of CLNM

**Figure 2:**
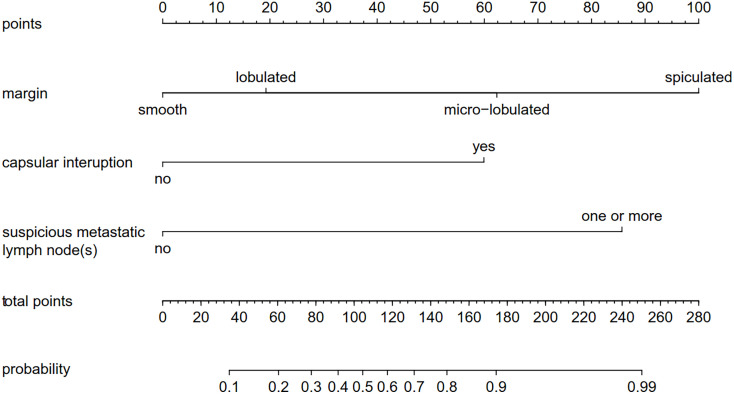
A nomogram forecasting the risk of CLNM for patients with MTC. The value of each of these variables was proportionally assigned a score based on the point scale. Subsequently, a total score could be obtained by summing up each subject’s score and identifying it on the lower total point scale. The probability of CLNM in each patient can finally be determined. CLNM, cervical lymph node metastasis; MTC, medullary thyroid carcinoma.

**Figure 3:**
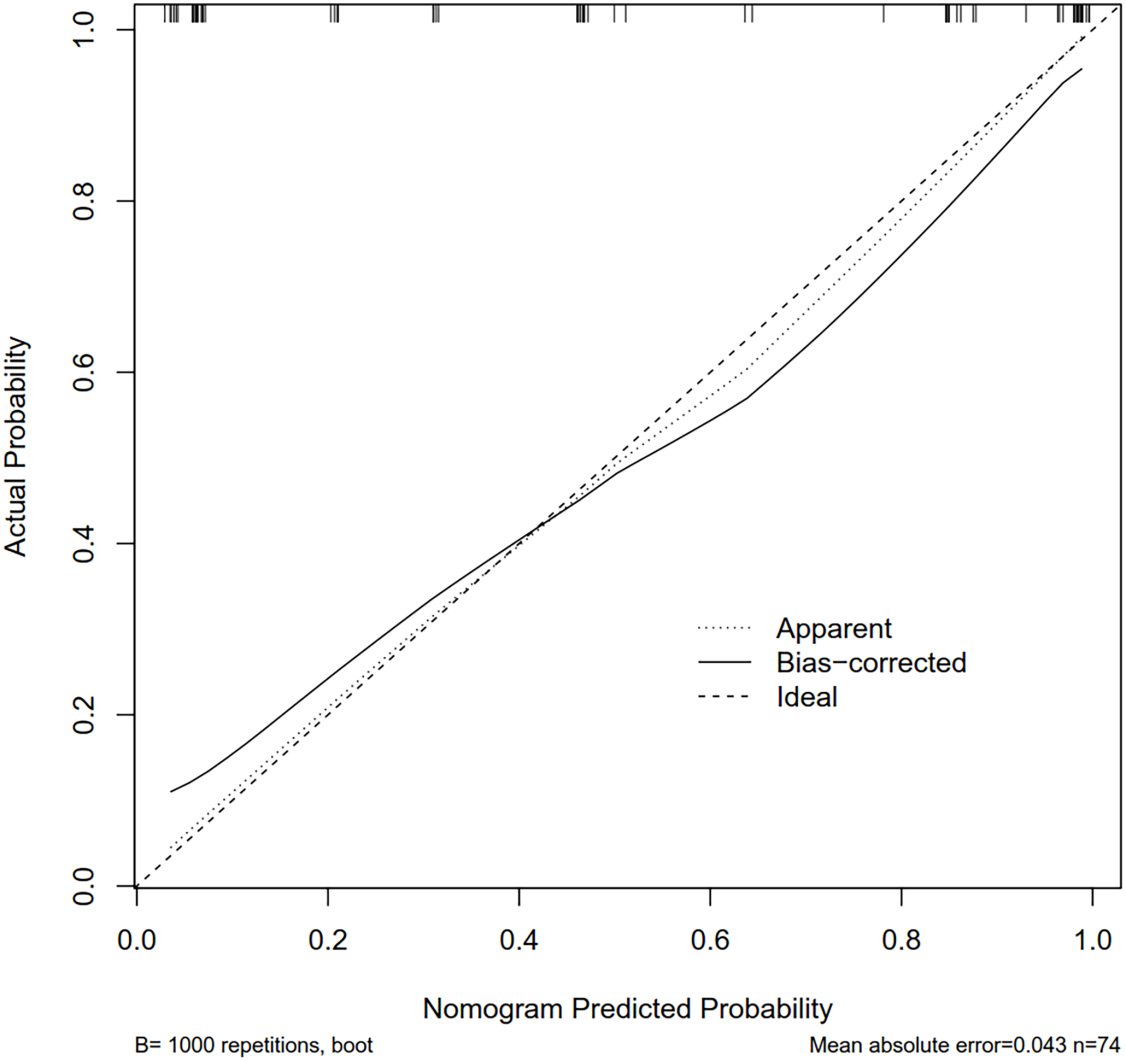
The calibration curves for the nomogram. The x-axis represents the nomogram-predicted CLNM probabilities, and y-axis represents the actual probability of CLNM. Perfect prediction would be along the 45-degree line. The solid curve is bias-corrected by bootstrapping (B = 1,000 repetitions), indicating the observed nomogram performance. CLNM, cervical lymph node metastasis; MTC, medullary thyroid carcinoma.

A nomogram was built based on the results of the binary logistic regression that integrated the above three independent preoperative suspicious features for predicting CLNM of the MTC (**Figure 2**). The value of each of these variables (margin, extracapsular invasion, and US-reported LN status) was proportionally assigned a score based on the point scale. Subsequently, a total score could be obtained by summing up each subject’s score and identifying it on the lower total point scale, and the probability of CLNM in each patient can be finally determined. According to the ROC analysis, the nomogram exhibited good discrimination, with an area under the ROC curve of 0.919 (95% CI, 0.856–0.932). A calibration curve of the nomogram exhibits that the predicted value is in good agreement with the actual probability of CLNM with additional 1,000 bootstraps (**Figure 3**).

### An example of the nomogram in use

**Figure 4:**
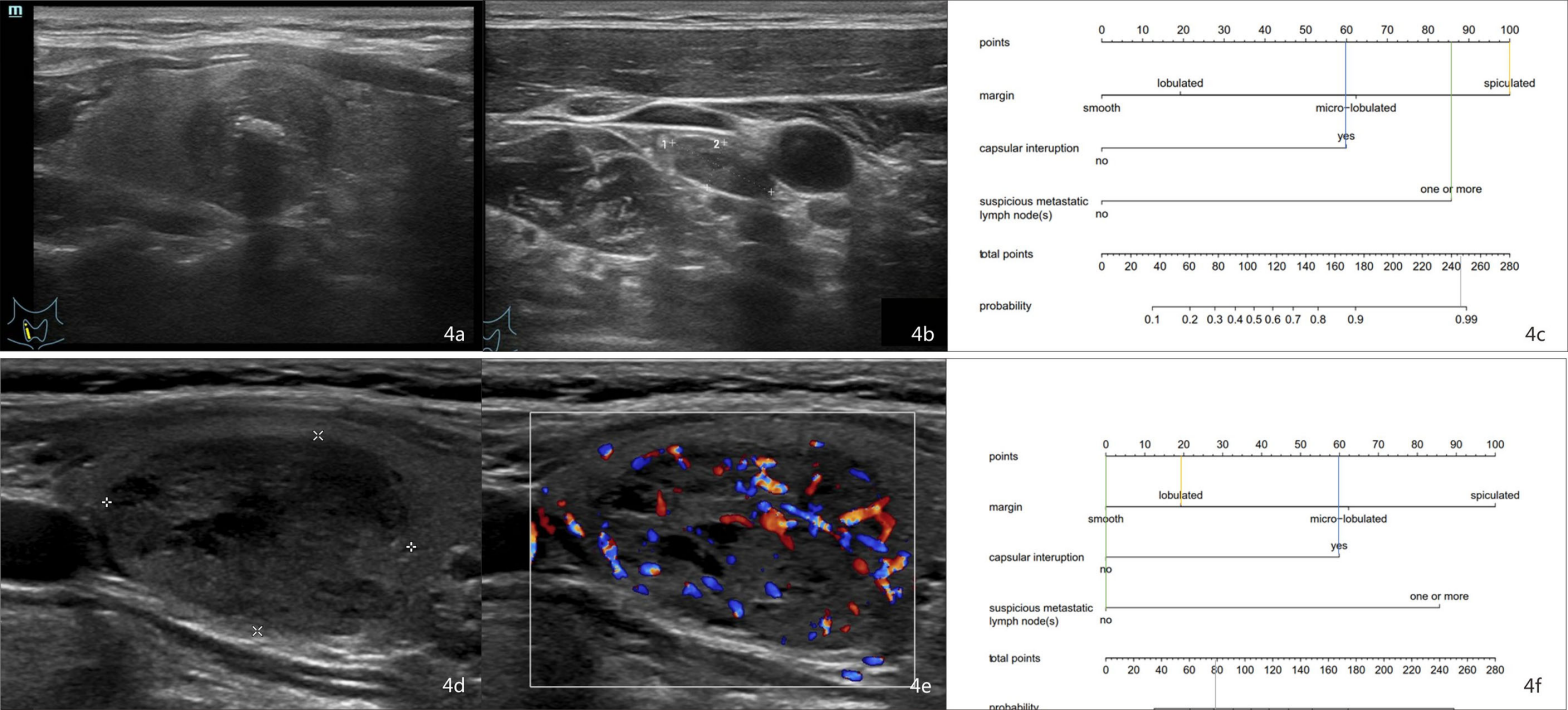
Examples of using the nomogram to predict the individual probability of CLNM by drawing straight lines across the diagram. First, draw lines straight upward to the point axis for each factor (margin status, orange line; capsular interruption, blue line; US-reported LN status, green line). Then, calculate total points for each of the predictors. Finally, draw a line (the gray line) straight down to the “Total points” scale to obtain the “Risk” of CLNM. **(A)** A 54-year-old man with MTC. **(B)** A small (size, 1.28 * 0.55 cm) lymph node was discovered, so the score was 85. The margin is spiculated, so the score was 100. The capsule is interrupted, so the score was 60. **(C)** The total point was 245 (100 + 60 + 85). The nomogram displayed that the chance of CLNM was more than 98%. Postoperative histology proved the positive CLNM. **(D, E)** A 60-year-old woman with MTC. The margin is lobulated, so the score was 20. The capsule is interrupted, so the score was 60. No suspicious lymph node was discovered, so the score was 0. The total point was 80 (20 + 60 + 0). **(F)** The probability of CLNM was approximately 30% by projecting a line straight down on the “Total points” scale. Postoperative pathology revealed that the patient has negative CLNM. CLNM, cervical lymph node metastasis; MTC, medullary thyroid carcinoma.

For example, the risk of CLNM in patient 1 who has a lesion in the right thyroid lobe with spiculated margins, extracapsular invasion, and US-reported suspicious LN (**Figures 4A, B**) could be calculated to be 98% by drawing a vertical line on the “Total points” scale (**Figure 4C**). Pathology proves the positive CLNM. In patient 2 who has a nodule in the right lobe with lobulated margins, no US-reported suspicious LNs, and no extracapsular invasion (**Figures 4D, E**), the risk of CLNM turned out to be about 30% (**Figure 4F**). Postoperative pathology demonstrated the negative CLNM, although she had a high serum Ctn (>2,000 pg/ml).

## Discussion

There are no evident recommendations for deciding the extent of LND in MTC patients with no clinical evidence of CLNM in preoperative images, so the clinical management of these patients can be challenging. Supporters, for example, Al-Qurayshi et al. (17), pointed out that preventive neck dissection upgraded 17.7% and 14.3% of patients to N1A and N1b. A meta-analysis (18) showed that LND was associated with lower mortality, suggesting that preventive LND is beneficial. However, it raises the risk of potential surgical complications such as recurrent laryngeal nerve injury, chylorrhea, and reduced parathyroid function (9). Therefore, an accurate and convenient way to directly assess the preoperative risk of CLNM is urgently needed. Machens et al. (19) found that about 70% of MTC patients with CCLNM had LCLNM, which suggests that for MTC patients with CCLNM, preventive resection of ipsilateral cervical LNs is necessary. The National Comprehensive Cancer Network (NCCN) guidelines suggest that LND should be performed when the primary tumor is more than 1 cm or there is CCLNM (7). Therefore, our research focuses on the related factors of CLNM rather than LCLNM or CCLNM in patients with MTC, so as to provide a basis for preventive LND. Oh et al. (12) indicate that high preoperative Ctn levels (>65 pg/ml) and a larger tumor size (>1.5 cm), irregular shape, spiculated margin, and subcapsular location of the tumor in preoperative neck US are significantly associated with LCLNM of MTCs. MTCs with two or more predictors are at higher risk for LCLNM. MTCs with fewer than two predictors have a very low probability of LCLNM and might be suitable for treatment without prophylactic lateral LND. However, the equal split of the variables in the work of Oh et al. is quite impractical considering the nonlinear relationship between the variable and LCLNM that brought forward further questions about the representativeness of their prediction model for other populations.

The nomogram is a predictive tool that uses a visual chart of a statistical predictive model to solve the complexity of balancing different variables; it also reduces the bias caused by individual abnormal clinical or imaging variables. Moreover, nomograms play important roles in personalized risk stratification and help doctors in choosing the treatments when no guidelines exist, especially in the field of cancer (20). Previous studies (21–24) have demonstrated that nomograms have been useful in predicting CLNM risk in PTC patients, but there were relatively few reports for predicting CLNM risk in MTC patients. In our present study, a noninvasive nomogram model for the preoperative prediction of CLNM of MTC was built using US features. This nomogram model exhibited a satisfying result with a good discriminative ability of a C-index of 0.919 (95% CI, 0.856–0.932) and a good calibration.

According to our findings, tumor margin, US-reported suspicious LNs, and extracapsular invasion were independent risk factors for CLNM of MTC. The presence of ETE in MTC is considered a risk factor for aggressive behavior and CLNM (25–27). The extrathyroidal extension (ETE) is confirmed by postoperative pathology. However, extracapsular invasion, defined as a tumor abutting the thyroid capsule or there was a discontinuity of the capsule, could be a useful preoperative US feature for predicting ETE in pathological reports (28). MTC cancer cells are low-differentiated, highly invasive, and often grow infiltrating. The thyroid capsule can be regarded as a barrier. Once the malignant tissue infiltrates the capsule, it is easy to enter the lymphatic circulation system, which makes it extremely prone to LN and distant metastasis (29). In this study, in MTC patients with CLNM, the invasion of the capsule was significantly higher than that of noninvasive (80.5% vs 18.2%), which indicates that the invasion of the capsule has a greater impact on CLNM. This finding suggests the importance of careful US examination to determine extracapsular invasion of MTCs because of its usefulness in predicting CLNM.

The finding that merits discussion is the potential influence of the tumor margin on CLNM, which is the largest contributor to scores of the US-based model. A lobulated or spiculated margin on a preoperative US image was considered a predictive factor for CLNM in MTC. With significant improvements of ultrasonic resolution and the application of higher-frequency probes, margin details of thyroid nodules are better exhibited now, which can promote a more nuanced assessment of thyroid lesions. A smooth margin of the tumor was almost found in the CLNM-negative group. When the MTCs present expansile growth, a group of tumor cells “pushing” into surrounding normal thyroid tissue, forming a lobulated margin, this result was consistent with those earlier studies (30–33). When the MTC infiltrated and grew extensively, the boundary presents microlobulated or spiculated, and it was more likely to infiltrate the thyroid capsule and metastasize to adjacent cervical LNs. In this study, CLNM risk increased with the microlobulated or spiculated margin. Therefore, attention should be fixed on the identification of margins.

It is reported that neck ultrasonography demonstrated low sensitivity but high specificity and a positive rate in diagnosing CLNM. Neck US showed only a 6% sensitivity when diagnosing CCLNM (34). Especially, micrometastasis may be hidden by the thyroid tissues. In our study, preoperative US examination found suspicious LNs in 29 cases (29/41, 70.7%) with a relatively low sensitivity (65.9%) but high specificity (93.9%) and positive predictive value (PPV) (93.1%), higher than those in previous studies (85%–88% and 77%–83%, respectively) (35).

In our present study, univariate analysis indicated that the level of preoperative serum Ctn and tumor’s size were obviously higher and larger in patients with positive CLNM than those with negative CLNM, which agreed with the previous research that a high preoperative Ctn level is related to the extent of CLNM and poor prognosis in MTC (36–41). However, multivariate analysis showed that they were not independent predictors for predicting CLNM, which may be due to the small sample size; the other reason may be that the level of preoperative serum Ctn and the size of MTC were partially overlapped. With the increase in the diameter of the primary tumor, the basal Ctn level gradually increases, as does the number of LNMs. Therefore, the two factors are strongly correlated.

This study successfully constructed an US-based nomogram, which perfectly stratified patients according to their risk of CLNM and demonstrated a satisfactory performance. We recommend that patients with high scores should undergo prophylactic LCLN dissection to prevent reoperations due to recurrence or metastasis. For patients with low scores, which indicate that they are at low risk of CLNM, prophylactic LCLN dissection should be avoided to reduce unnecessary damage and possible surgical complications. In the American Thyroid Association guidelines, prophylactic LCLN dissection based on the Ctn level is suggested with a Grade I recommendation (recommends neither for nor against it) (1). About 36% of the CLNM-negative patients of this study displayed high preoperative Ctn levels (>150 pg/ml) before the operation, which lead to the LCLN dissection. When the nomogram was used to evaluate the risk of CLNM in each patient, 75% of patients had low scores. This finding indicates that these MTC patients could choose a more suitable surgical strategy if the prediction model is used.

There are some limitations in our study. First, this is a retrospective single-center study that may be affected by selection biases. Second, our study failed to contain a complete biochemical assessment with serum Ctn, CEA levels, and other imaging studies. Third, it is worth noting that our nomograms have not been validated by external cohorts, and we will use other databases for calibration in our future studies.

In conclusion, we established and validated a user-friendly and accurate US-based nomogram for forecasting the probability of CLNM in MTC patients preoperatively, which may guide clinicians in stratifying patients and assist surgeons to choose the appropriate surgical strategy and thus reduce overtreatment of indolent MTC, which is suitable to the current trend toward personalized care.

## Data availability statement

The original contributions presented in the study are included in the article/supplementary material.. Further inquiries can be directed to the corresponding authors.

## Ethics statement

The studies involving human participants were reviewed and approved by the Ethics Committee of the Second Affiliated Hospital of Zhejiang University School of Medicine. Written informed consent from the participants’ legal guardian/next of kin was not required to participate in this study in accordance with the national legislation and the institutional requirements.

## Author contributions

ZL: Collection of data, interpretation of data and drafting the article; YH: Revising the article and provide the acquisition of funding; CY: Collection of data, interpretation of data; YW: Provide patient information and surgical specimens; QY: Provide pathological results; PH: general supervision of the research group. All authors contributed to the article and approved the submitted version.

## Funding

This work was supported by Natural Science Foundation nonprofit research projects of Zhejiang Province of China (LGF19H180020).

## Conflict of interest

The authors declare that the research was conducted in the absence of any commercial or financial relationships that could be construed as a potential conflict of interest.

## Publisher’s note

All claims expressed in this article are solely those of the authors and do not necessarily represent those of their affiliated organizations, or those of the publisher, the editors and the reviewers. Any product that may be evaluated in this article, or claim that may be made by its manufacturer, is not guaranteed or endorsed by the publisher.
